# Financial dynamics and post-pandemic recovery of a public multidisciplinary hospital in Kazakhstan: a five-year financial case study

**DOI:** 10.3389/fpubh.2026.1902342

**Published:** 2026-08-25

**Authors:** Maira Alimbetova, Kuralbai Kurakbayev, Zhumagali Ismailov, Rupal Najhawan, Zhanar Akimbaeva, Mairash Baimuratova, Marzhan Kassenova

**Affiliations:** 1 Department of Public Health and Social Sciences, Department of Health Economics and Medical Insurance, Kazakhstan’s Medical University “KSPH”, Almaty, Kazakhstan; 2 Caspian International School of Medicine, Almaty, Kazakhstan

**Keywords:** COVID-19, financial indicators, financial performance, hospital finance, Kazakhstan, public hospitals

## Abstract

**Background:**

The COVID-19 pandemic substantially affected the financial performance of healthcare organizations worldwide. However, evidence describing hospital financial dynamics during the pandemic and post-pandemic periods remains limited in Central Asia.

**Objective:**

To evaluate changes in the financial indicators of a public multidisciplinary hospital in Kazakhstan during 2020–2024 using audited financial statements. Methods: A retrospective quantitative case study was conducted using audited annual financial statements of the Almaty Multidisciplinary Clinical Hospital for 2020–2024. Horizontal, vertical, ratio, and trend analyses were performed to evaluate changes in assets, liabilities, receivables, inventories, fixed assets, liquidity, and financing structure. Long-term financial dynamics were additionally assessed using the coefficient of variation (CV) and compound annual growth rate (CAGR).

**Results:**

Marked changes in financial indicators were observed during the study period. Accounts receivable decreased from 463,934 thousand KZT in 2020 to 236,584 thousand KZT in 2021 before increasing to 434,491 thousand KZT in 2024. Inventories increased from 281,068 to 748,922 thousand KZT (CAGR = 27.8%). Accounts payable declined in 2021 and subsequently increased to 669,583 thousand KZT in 2024. The current liquidity ratio increased from 1.45 in 2020 to 1.76 in 2024, while the autonomy coefficient remained above 0.60 throughout the study period, indicating a consistently high proportion of equity in the hospital’s financing structure. Variability analysis showed that accounts payable exhibited the greatest dispersion (CV = 80.7%), whereas fixed assets remained the most stable financial component (CV = 6.9%).

**Conclusion:**

The financial indicators demonstrated substantial variation during the pandemic years, followed by a more stable pattern after 2022. The findings show that routinely available financial indicators can be used to monitor changes in hospital financial performance during periods of major healthcare system disruption. This study provides new evidence on hospital financial dynamics from Central Asia and may inform future comparative research on healthcare financing and organizational performance.

## 1 Introduction

Financial stability and liquidity are essential for maintaining continuity of care, ensuring access to medical services, and supporting the long-term functioning of healthcare organizations. Their importance becomes particularly evident in health systems where financing arrangements, reimbursement mechanisms, and institutional autonomy directly influence hospital operations and financial decision-making (1). Previous studies have shown that hospital financial performance is generally positively or neutrally associated with quality of care, whereas clearly negative associations are relatively uncommon (2). Consequently, financial indicators have become widely used instruments for evaluating organizational performance and supporting evidence-informed financial management in healthcare settings (3, 4).

Financial recovery has emerged as an increasingly important concept in health services research following the COVID-19 pandemic. Although no universally accepted definition exists, financial recovery is generally understood as the progressive restoration of a healthcare organization’s financial stability after a major external disruption. Rather than implying a complete return to pre-crisis conditions, financial recovery encompasses improvements in liquidity, financial flexibility, operating capacity, and the ability to sustain healthcare delivery under evolving economic circumstances (5, 6). In this context, financial indicators provide practical measures for assessing institutional adaptation over time, although they should be interpreted alongside operational and organizational factors (3).

The COVID-19 pandemic exposed substantial vulnerabilities in hospital financing worldwide. Sudden increases in demand for healthcare services, disruptions in routine medical care, emergency procurement of equipment and supplies, and modifications of reimbursement mechanisms placed considerable pressure on healthcare organizations. Many hospitals were required to maintain uninterrupted service delivery while adapting to rapidly changing operational and financial conditions. These challenges intensified interest in understanding how healthcare institutions manage liquidity, assets, liabilities, and financial recovery following large-scale system shocks. Beyond the immediate crisis response, understanding how hospitals recover financially after major disruptions has become an increasingly important area of health services research.

Evidence from different countries indicates that financial and operational performance in hospitals is influenced by a complex interaction of organizational and economic factors. For several acute conditions, higher levels of resource utilization have been associated with lower mortality rates, potentially reflecting greater access to advanced technologies and specialized care (7). Similarly, studies have demonstrated that patients treated in higher-resource hospitals may experience improved outcomes despite higher treatment costs (8). These findings suggest that financial capacity remains an important component of healthcare delivery, particularly in technologically intensive hospital settings.

Recent evidence suggests that post-pandemic recovery trajectories differ considerably across healthcare organizations. While some hospital systems demonstrated improvements in liquidity, operating performance, and financial stability after the acute phase of the pandemic, others continued to experience financial pressures related to rising labor costs, inflation, reimbursement constraints, and changing patterns of healthcare utilization (9, 10). Organizations with stronger financial reserves and more diversified financing structures generally demonstrated faster recovery and greater operational resilience, highlighting the importance of liquidity management and sustainable financing strategies in the post-pandemic period (9–11).

Organizational resilience has been described as the ability of healthcare organizations to anticipate, adapt to, and recover from major disruptions while maintaining essential functions (5). However, resilience is a multidimensional construct encompassing organizational, operational, financial, and managerial domains (12). Accordingly, financial indicators alone cannot directly measure organizational resilience but may provide indirect evidence of one of its important dimensions—financial adaptation following a system-wide shock. In the present study, organizational resilience is therefore considered an interpretive framework rather than a directly measured outcome.

Hospital financial performance also varies substantially across healthcare systems. In Europe, financial outcomes are shaped by ownership structures, governance models, and payment systems (13). In the United States, profitability is influenced by capital structure, indebtedness, workforce productivity, and organizational characteristics, while improvements in quality indicators are often accompanied by stronger financial performance (14, 15). Operational factors such as length of stay, bed utilization, workflow efficiency, and service volume contribute significantly to hospital expenditures and financial results (16). Administrative expenses account for a considerable share of healthcare spending and may influence overall organizational performance (17, 18). At the same time, standardization of administrative processes, digitalization, and organizational reforms have demonstrated the potential to reduce administrative costs and improve efficiency (19–22).

Following the acute phase of the pandemic, attention has increasingly shifted toward financial recovery and institutional adaptation. Liquidity management, debt obligations, capital investment, and asset renewal have emerged as important indicators of a hospital’s capacity to restore routine operations and maintain service delivery under changing economic conditions. International studies have documented substantial variation in recovery trajectories across healthcare organizations, reflecting differences in financing structures, governance arrangements, resource availability, and broader macroeconomic conditions (23–31).

Despite growing international evidence, empirical research on hospital financial recovery remains limited in Central Asia. This gap is particularly relevant for Kazakhstan, where public healthcare organizations operate within an evolving healthcare financing and reimbursement system and must simultaneously maintain service continuity, preserve financial sustainability, and modernize infrastructure under constrained resources. In addition to recovering from the unprecedented disruptions caused by the COVID-19 pandemic, healthcare institutions have had to adapt to ongoing changes in financing and governance, making the interpretation of post-pandemic financial performance increasingly complex. Understanding how hospitals restore financial stability during this period is therefore important for both institutional management and broader health system planning.

The present study examines the financial dynamics of the State Communal Enterprise on the Right of Economic Management “Almaty Multidisciplinary Clinical Hospital” (SCE REM AMCH), a public multidisciplinary hospital operating under a public-private partnership (PPP) arrangement in Kazakhstan, during 2020–2024. Using annual financial statements and institutional operational indicators, the study evaluates changes in assets, liabilities, liquidity, financing structure, and selected operational characteristics across the pandemic and post-pandemic periods.

The objective of this study was to evaluate changes in assets, liabilities, liquidity, financing structure, and selected operational indicators of a public multidisciplinary hospital in Kazakhstan during the COVID-19 pandemic and the subsequent post-pandemic period (2020–2024), in order to characterize its financial recovery trajectory.

## 2 Materials and methods

This retrospective quantitative case study examined the financial dynamics of a public multidisciplinary hospital in Kazakhstan during the period 2020–2024. The analysis was based on official annual financial statements of the State Communal Enterprise on the Right of Economic Management “Almaty Multidisciplinary Clinical Hospital” (SCE REM AMCH), a major provider of specialized inpatient care in Almaty. The study utilized audited annual financial reports prepared in accordance with the accounting regulations and reporting requirements of the Republic of Kazakhstan. Two principal reporting documents were included:

- Balance sheet (Form No. 1), containing information on assets, liabilities, equity, and long-term financing sources;
- Income statement (Form No. 2), providing information on operating revenues, expenditures, and government funding received during the reporting period.

The study was designed as a retrospective longitudinal financial case study. The analysis was descriptive and aimed to characterize financial changes during the COVID-19 pandemic and the subsequent recovery period. The study was not intended to evaluate causal relationships or the independent effects of the public-private partnership model.

All financial values were reported in thousand Kazakhstani tenge (KZT). Financial indicators were analyzed in nominal terms because the primary objective of the study was to assess structural changes, financial dynamics, and organizational recovery patterns over time. The dataset was complete for all study years and did not contain missing observations.

Quarterly financial statements were not publicly available for the institution. Therefore, annual audited financial reports represented the highest level of accessible financial reporting and served as the primary data source for the study.

### 2.1 Study setting

Almaty Multidisciplinary Clinical Hospital is a public multidisciplinary healthcare institution providing specialized and high-technology medical services to residents of Almaty and surrounding regions. Since 2020, the hospital has operated under a public-private partnership model. Within this framework, the institution continues to provide publicly funded healthcare services while functioning under a mixed governance arrangement that combines public ownership with private-sector participation in management and development activities. This organizational context is relevant for interpreting the hospital’s financial trajectory during the pandemic and post-pandemic periods. The PPP arrangement was considered the institutional context of the study rather than the intervention under evaluation.

The hospital provides care in cardiology, neurosurgery, cerebrovascular diseases, acute coronary syndromes, and other complex clinical conditions requiring advanced diagnostic and therapeutic capabilities. The institution operates 360 inpatient beds, including specialized cardiology, stroke, and intensive care units, and delivers both emergency and planned medical services on a continuous basis.

The workforce includes approximately 220 physicians, more than 60% of whom hold the highest professional qualification category, supported by approximately 450 nursing personnel and 330 junior healthcare workers. According to institutional reports, more than 36,000 patients received medical care in 2023, including over 13,000 inpatient admissions and approximately 7,000 surgical procedures. Given its scale, service profile, and role within the regional healthcare system, the hospital provides an informative setting for examining financial dynamics during the COVID-19 pandemic and subsequent recovery period.

### 2.2 Study variables

The analysis focused on key financial indicators describing changes in the hospital’s financial position and balance-sheet structure during the study period. The following categories of indicators were evaluated:

- Asset indicators: accounts receivable, inventories, fixed assets, and total assets.
- Liability indicators: current liabilities, long-term liabilities, total liabilities, and equity.
- Liquidity indicators: current liquidity ratio (CLR).
- Capital structure indicators: liability coverage ratio (LCR), autonomy coefficient (AC), and debt-to-assets ratio.
- Growth and variability indicators: year-to-year growth rates, compound annual growth rate (CAGR), coefficient of variation (CV), and indexed values (2020 = 100).

These indicators were selected because they represent commonly used measures for describing financial position, liquidity, financing structure, and temporal changes in institutional financial performance. In the present study, financial recovery was operationalized as changes in these financial indicators over time rather than as a direct measure of organizational performance.

### 2.3 Analytical approach

The analytical framework combined complementary descriptive methods commonly applied in financial and health services research.

#### 2.3.1 Horizontal analysis

Horizontal analysis was performed to evaluate year-to-year changes in the principal financial indicators. Absolute values and annual percentage changes were calculated for major asset, liability, and equity categories.

#### 2.3.2 Vertical analysis

Vertical analysis was used to examine the composition of assets, liabilities, and equity across the study period and to describe changes in the hospital’s balance-sheet structure.

#### 2.3.3 Financial ratio analysis

Financial ratio analysis was conducted using audited annual financial statements. The analysis included the current liquidity ratio, liability coverage ratio, autonomy coefficient, and debt-to-assets ratio. Complete audited financial and operational data for 2018–2024 are provided in Supplementary Tables S1–S3. Supplementary Table S1 presents annual balance-sheet indicators, Supplementary Table S2 summarizes annual income statement data, and Supplementary Table S3 provides annual operational indicators. Consistent with the predefined study design, the primary analyses presented in this study were restricted to the 2020–2024 period.

#### 2.3.4 Trend analysis

Multi-year trend analysis was performed to describe temporal changes in financial indicators between 2020 and 2024. Particular attention was given to differences between the early pandemic period (2020–2021) and the subsequent observation period (2022–2024).

### 2.4 Financial indicators

Table 1 summarizes the principal financial indicators included in the analysis.

**Table 1:** Financial indicators included in the study.

Category	Description
Growth indicators	Annual percentage changes, compound annual growth rate, and indexed values (2020 = 100).
Liquidity indicators	Current liquidity ratio (CLR), describing the hospital’s short-term liquidity position.
Capital structure indicators	Liability coverage ratio (LCR), autonomy coefficient (AC), and debt-to-assets ratio, describing the relationship between assets, liabilities, and equity.
Variability indicators	Mean, standard deviation (SD), and coefficient of variation (CV) describing long-term variability of financial indicators.

The following financial ratios were calculated:

- Current Liquidity Ratio (CLR)

CLR = Current Assets/Current Liabilities

- Liability Coverage Ratio (LCR)

LCR = (Equity + Long-Term Liabilities)/Total Liabilities

- Autonomy Coefficient (AC)

AC = Equity/Total Assets

- Debt-to-Assets Ratio

DAR = Total Liabilities/Total Assets.

Where:

CA = Current Assets;

CL = Current Liabilities;

TA = Total Assets;

TL = Total Liabilities;

EQ = Equity;

LTL = Long-term liabilities.

Financial ratios were used to describe changes in liquidity and financing structure throughout the study period. Because publicly available financial reports contained limited detail for certain balance-sheet components, the calculated ratios were interpreted primarily as descriptive indicators of temporal changes rather than as comprehensive measures of institutional financial performance.

### 2.5 Statistical analysis

Descriptive statistical methods were used to summarize annual financial indicators. Results are presented as absolute values, annual percentage changes, financial ratios, coefficients of variation, compound annual growth rates, indexed values (2020 = 100), and multi-year trends.

Given the limited number of annual observations and the descriptive objective of the study, inferential statistical analyses were not performed. The coefficient of variation was calculated using the sample standard deviation. CAGR and indexed values were calculated to summarize long-term changes in the principal financial indicators and are presented as descriptive measures of financial dynamics.

Financial indicators were analyzed in nominal terms because the primary objective was to evaluate structural accounting changes and temporal financial dynamics rather than estimate inflation-adjusted economic growth.

All statistical analyses and data management procedures were performed using Microsoft Excel 365 and IBM SPSS Statistics version 26.0.

### 2.6 Ethical considerations

The study was based exclusively on aggregated institutional financial records and did not involve human participants, personal health information, or confidential patient data. In accordance with national regulations governing secondary analyses of administrative and financial information, ethical review board approval and informed consent were not required.

## 3 Results

### 3.1 Dynamics of assets, liabilities, and funding structure (2020–2024)

The financial data presented in Table 2, expressed uniformly in thousand KZT, demonstrate substantial changes in the hospital’s assets, liabilities, and equity during the study period (2020–2024). Complete audited financial and operational data for 2018–2024 are provided in Supplementary Tables S1–S3. The most pronounced fluctuations were observed during 2020–2021, whereas financial indicators showed comparatively smaller year-to-year changes during the subsequent observation period.

**Table 2:** Dynamics of key financial indicators, 2020–2024 (thousand KZT).

Year	Accounts receivable	Inventories	Fixed assets	Total assets	Current liabilities	Long-term liabilities	Total liabilities	Equity
2020	463,934	281,068	3,032,827	3,875,083	1,002,790	519,969	1,522,759	2,352,324
2021	236,584	597,809	2,665,257	3,875,026	766,372	637,216	1,403,543	2,471,438
2022	198,455	655,870	2,675,983	3,626,449	770,341	437,519	1,207,860	2,418,589
2023	338,180	665,253	2,618,221	3,733,221	644,691	437,519	1,082,210	2,651,011
2024	434,491	748,922	2,970,876	4,380,707	1,294,533	432,961	1,727,495	2,653,212

Accounts receivable declined from 463,934 thousand KZT in 2020 to 236,584 thousand KZT in 2021 (−49.0%) and further to 198,455 thousand KZT in 2022. Thereafter, accounts receivable increased to 338,180 thousand KZT in 2023 and reached 434,491 thousand KZT in 2024, approaching the baseline level observed in 2020.

Inventories increased from 281,068 thousand KZT in 2020 to 597,809 thousand KZT in 2021 and continued to rise throughout the study period, reaching 748,922 thousand KZT in 2024, corresponding to an overall increase of approximately 2.7-fold compared with baseline.

Fixed assets decreased from 3,032,827 thousand KZT in 2020 to 2,665,257 thousand KZT in 2021, remained relatively stable during 2022–2023, and increased to 2,970,876 thousand KZT in 2024.

Current liabilities declined between 2020 and 2023 before increasing in 2024. Long-term liabilities remained comparatively stable throughout the study period, ranging from 432,961 to 637,216 thousand KZT. Total liabilities decreased from 1,522,759 thousand KZT in 2020 to 1,082,210 thousand KZT in 2023, followed by an increase to 1,727,495 thousand KZT in 2024. Equity showed comparatively limited variation over time, ranging from 2,352,324 thousand KZT in 2020 to 2,653,212 thousand KZT in 2024.

Overall, the balance-sheet data indicate substantial temporal variation in several financial indicators during the study period, while fixed assets, long-term liabilities, and equity remained comparatively stable relative to other balance-sheet components.

### 3.2 Analysis of growth rates and operational efficiency

Year-to-year analysis of the principal balance sheet components demonstrated substantial changes in the hospital’s financial position over the study period (Table 3). Accounts receivable declined by 49.0% in 2021 and by a further 16.1% in 2022, followed by recovery in 2023 (+70.4%) and continued growth in 2024 (+28.5%). This pattern indicates considerable variation in outstanding receivables across the observation period.

**Table 3:** Year-to-year changes in key financial indicators (%).

Indicator	2020 → 2021	2021 → 2022	2022 → 2023	2023 → 2024
Accounts receivable	−49.0	−16.1	+70.4	+28.5
Inventories	+112.7	+9.7	+1.4	+12.6
Fixed assets	−12.1	+0.4	−2.2	+13.5
Total assets	0.0	−6.4	+2.9	+17.3
Current liabilities	−23.6	+0.5	−16.3	+100.8
Long-term liabilities	+22.5	−31.3	0.0	−1.0
Total liabilities	−7.8	−13.9	−10.4	+59.6
Equity	+5.1	−2.1	+9.6	+0.1

Inventories increased continuously throughout the study period. The largest increase occurred in 2021 (+112.7%), whereas subsequent annual growth remained moderate, ranging from 1.4 to 12.6%. Fixed assets decreased during 2021 (−12.1%), remained relatively stable during 2022–2023, and increased by 13.5% in 2024.

Total assets remained relatively stable between 2020 and 2023, with annual changes ranging from −6.4 to +2.9%, followed by a marked increase of 17.3% in 2024. Total liabilities decreased progressively between 2020 and 2023 before increasing by 59.6% in 2024, primarily owing to higher current liabilities. Equity remained comparatively stable throughout the study period, with annual changes ranging from −2.1 to +9.6%.

Overall, the financial dynamics indicate gradual changes in the hospital’s balance-sheet structure throughout the observation period.

### 3.3 Liquidity and financing structure indicators

The liquidity and financing structure indicators calculated from the audited financial statements are presented in Table 4. The current liquidity ratio increased from 1.45 in 2020 to 1.76 in 2024, suggesting gradual improvement in the hospital’s capacity to meet short-term obligations. The liability coverage ratio also increased over the study period, indicating higher long-term coverage of liabilities. The autonomy coefficient remained relatively stable, with equity consistently accounting for more than 60% of total assets. Consistent with this pattern, the debt-to-assets ratio declined from 0.39 in 2020 to 0.29 in 2023 before increasing to 0.39 in 2024, reflecting changes in the proportion of assets financed by liabilities. Overall, the financial ratios indicate gradual stabilization of the hospital’s financial position during the post-pandemic period.

**Table 4:** Financial ratios.

Year	Current liquidity ratio (CLR) current assets/current liabilities	Liability coverage ratio (LCR) (equity + long-term liabilities)/total liabilities	Autonomy coefficient (AC) equity/total assets	Debt-to-assets ratio total liabilities/total assets
2020	1.45	1.89	0.61	0.39
2021	1.62	2.22	0.64	0.36
2022	1.68	2.37	0.67	0.33
2023	1.72	2.85	0.71	0.29
2024	1.76	1.79	0.61	0.39

CLR, current liquidity ratio; LCR, liability coverage ratio; AC, autonomy coefficient.

**Figure 1:**
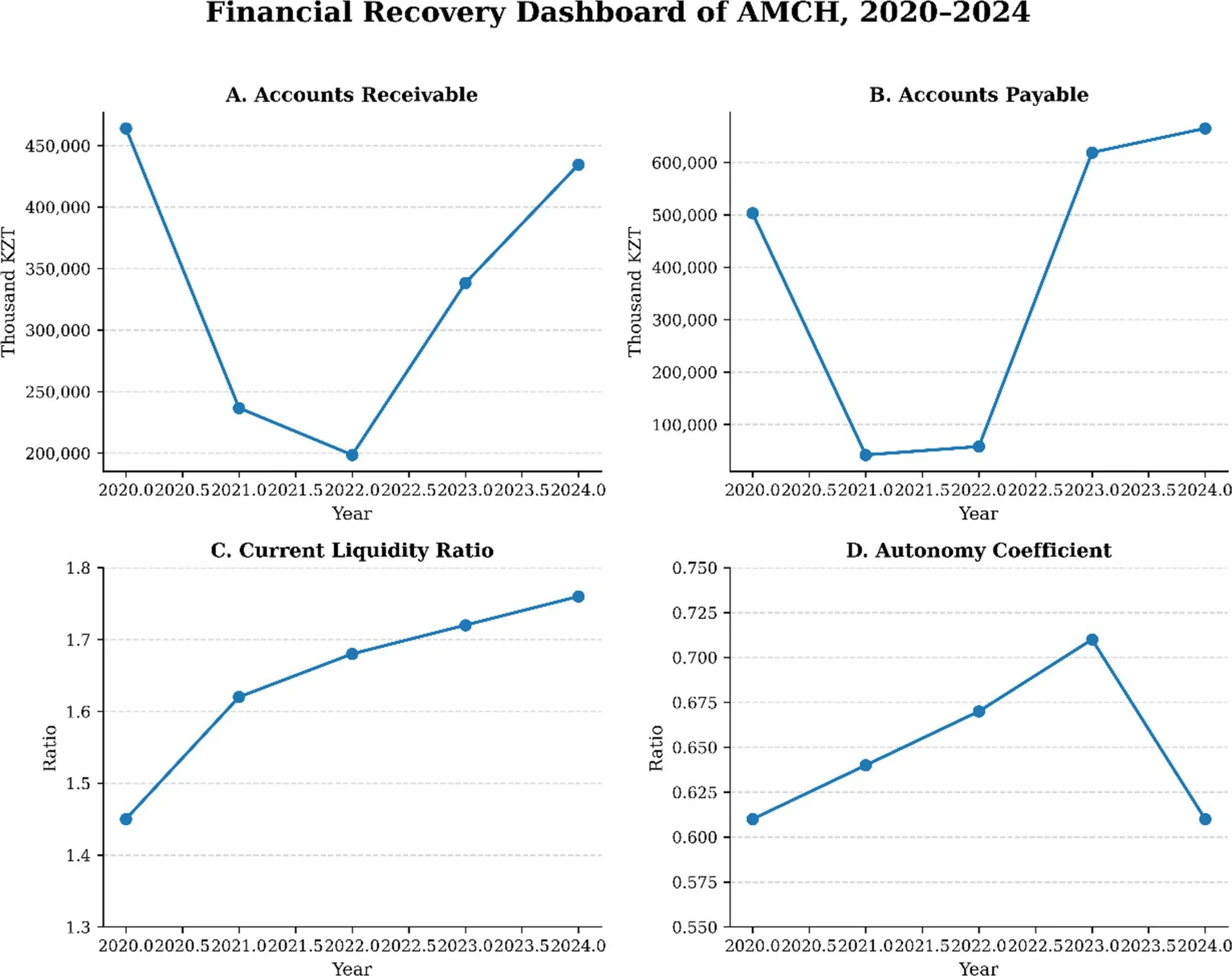
Trends in selected financial indicators of Almaty Multidisciplinary Clinical Hospital, 2020-2024. **(A)** Accounts receivable; **(B)** accounts payable; **(C)** current liquidity ratio; **(D)** autonomy coefficient.

The temporal evolution of the hospital’s principal financial indicators is presented in Figure 1. Panel A shows changes in accounts receivable over the study period. Panel B illustrates fluctuations in accounts payable, with a marked increase after 2022. Panel C demonstrates the gradual increase in the current liquidity ratio throughout 2020–2024. Panel D presents changes in the autonomy coefficient, reflecting the proportion of total assets financed by equity. Overall, the indicators demonstrate changes in the hospital’s financial position during the pandemic and post-pandemic periods, with generally more stable financial indicators observed after 2022.

Collectively, the indicators demonstrate gradual changes in the hospital’s financial position over the study period.

### 3.4 Variability and Long-term dynamics of financial indicators

To characterize the variability and long-term dynamics of the hospital’s financial indicators, the coefficient of variation, calculated using the sample standard deviation, and the compound annual growth rate were estimated using five annual observations (2020–2024). In addition, indexed values (2020 = 100) were calculated to facilitate comparison of long-term changes across indicators (Table 5).

**Table 5:** Long-term dynamics and variability of financial indicators, 2020–2024.

Indicator	Mean	SD	CV (%)	CAGR (%)	2024 Index (2020 = 100)
Accounts receivable (thousand KZT)	334,329	117,112	35.0	−1.6	93.7
Accounts payable (thousand KZT)	380,546	306,960	80.7	6.8	130.2
Inventories (thousand KZT)	589,784	180,802	30.7	27.8	266.5
Fixed assets (thousand KZT)	2,792,633	193,465	6.9	−0.5	98.0

SD, standard deviation; CV, coefficient of variation (calculated using the sample standard deviation); CAGR, compound annual growth rate; 2024 index (2020 = 100) = (value in 2024/value in 2020) × 100.

Accounts payable demonstrated the highest variability during the study period (CV = 80.7%), whereas fixed assets showed the lowest variability (CV = 6.9%), indicating a comparatively stable capital asset base throughout the observation period. Accounts receivable and inventories exhibited intermediate levels of variability, with CV values of 35.0 and 30.7%, respectively.

Indexed trend analysis showed that inventories experienced the largest relative increase, reaching an index value of 266.5 in 2024 compared with the 2020 baseline. Accounts payable also increased over the study period (index = 130.2), whereas accounts receivable declined slightly (index = 93.7). Fixed assets remained close to their baseline level (index = 98.0), indicating minimal long-term change. Consistent with these findings, CAGR values demonstrated the strongest long-term growth for inventories (27.8%), positive growth for accounts payable (6.8%), and only marginal changes for accounts receivable (−1.6%) and fixed assets (−0.5%).

Given the limited number of annual observations, both CV and CAGR should be interpreted as descriptive measures of temporal variation rather than inferential indicators of financial performance.

## 4 Discussion

### 4.1 Pandemic-related disruption of hospital financial flows

The financial changes observed during 2020–2021 indicate that the pandemic period was accompanied by substantial shifts in the hospital’s balance-sheet structure. The marked decline in accounts receivable and the concurrent increase in inventories occurred during the period of the COVID-19 emergency, when healthcare systems worldwide experienced unprecedented organizational and financial disruption. Similar changes have been described in other countries, where emergency payment mechanisms, centralized procurement, and temporary modifications of reimbursement systems altered routine financial processes and affected hospital balance sheets (25, 26).

A comparable pattern was observed for accounts payable and inventories. In the present study, inventories increased markedly while accounts payable declined during the same period. Although the available financial statements do not allow the underlying causes of these changes to be determined, similar trends have been reported in hospitals facing disruptions in supply chains, shortages of essential medical products, and rapidly changing service demands during the pandemic (27, 28). Previous studies also suggest that maintaining larger inventories became a common organizational strategy to reduce the risk of supply interruptions and support continuity of care under uncertain conditions (29, 30).

These findings should therefore be interpreted within the broader context of the pandemic rather than as isolated accounting events. At the same time, because the present study was based exclusively on annual financial reports, it cannot determine the relative contribution of specific financing policies, procurement practices, or managerial decisions to the observed changes.

### 4.2 Recovery of operational financing and liquidity

From 2022 onward, most financial indicators showed a more stable pattern than during the first 2 years of observation. Accounts receivable increased gradually after 2022, while liquidity indicators remained favorable throughout the study period. By 2024, several balance-sheet indicators had approached levels comparable with those observed at the beginning of the study period, suggesting progressive stabilization of the hospital’s financial position.

The current liquidity ratio increased from 1.45 in 2020 to 1.76 in 2024, indicating an improved capacity to meet short-term obligations. Similar trends have been reported in several OECD countries, where hospitals experienced gradual improvements in financial indicators following the acute phase of the pandemic (31). Although the mechanisms underlying these changes were beyond the scope of the present study, previous reports have linked post-pandemic improvements in liquidity with broader recovery of healthcare activity and greater organizational flexibility (32).

These observations may be relevant for Kazakhstan and other middle-income countries undergoing healthcare financing reforms. Stable liquidity and balanced financial management are important for maintaining continuity of healthcare services, particularly in hospitals providing specialized and high-technology care (33). At the same time, the findings should not be interpreted as evidence of a complete financial recovery, since the study evaluated descriptive financial indicators rather than the broader organizational or clinical performance of the institution.

The present findings are also consistent with international reports emphasizing that resilient healthcare systems require not only the capacity to respond during public health emergencies but also mechanisms that support financial stability in the years following major system-wide disruptions (34).

### 4.3 Financial autonomy and institutional resilience

One of the notable findings of this study was the relatively stable financing structure observed throughout the study period despite substantial fluctuations in several balance-sheet indicators. The autonomy coefficient remained above 0.60 during all years of observation, indicating that equity consistently represented a substantial proportion of the hospital’s total assets. Although accounts receivable, inventories, and liabilities changed considerably between 2020 and 2024, the overall capital structure remained comparatively stable.

Previous studies suggest that healthcare organizations with stronger financial and managerial autonomy may be better positioned to respond to changing operating conditions because greater flexibility can facilitate resource allocation and adaptation to evolving service demands (35, 36). However, the present study was not designed to examine the relationship between financial autonomy and organizational performance. Therefore, these findings should be interpreted as descriptive evidence of the hospital’s financing structure rather than as proof of a direct association with institutional resilience.

Maintaining an appropriate balance between equity and liabilities remains an important consideration for publicly funded hospitals. Excessive reliance on external financing may increase financial vulnerability during periods of economic uncertainty, whereas a stronger equity position may contribute to greater long-term financial stability (37). This issue is particularly relevant in Kazakhstan, where public healthcare organizations operate within a mixed financing system that combines state budget funding with payments from the Compulsory Social Health Insurance system.

The hospital included in this study operated under a public-private partnership model throughout the observation period. Although this organizational arrangement may have influenced financial management and resource allocation, the present study was not designed to evaluate the impact of the governance model itself. Consequently, the observed financial patterns should not be interpreted as evidence of the effectiveness of public-private partnerships. Comparative studies involving hospitals with different organizational models are needed to clarify whether governance structure contributes to differences in financial performance and long-term institutional sustainability.

### 4.4 Capital investment and post-pandemic modernization

The increase in fixed assets observed in 2024 indicates renewed capital investment following the earlier decline recorded in 2021. Because the available financial statements do not contain detailed information on individual capital projects, the specific factors underlying this increase cannot be determined from the present analysis. Nevertheless, similar patterns have been described in healthcare systems where investment in equipment and infrastructure resumed after the acute phase of the COVID-19 pandemic.

Previous studies have consistently reported that investment in healthcare infrastructure and medical equipment is associated with improved service capacity, operational efficiency, and quality of care (38, 39). In addition, hospitals that continue capital investment during periods of system recovery may restore service capacity more effectively than institutions in which modernization is substantially delayed (40).

At the same time, investment in infrastructure is associated with continuing financial commitments. International experience indicates that expansion of capital assets is frequently accompanied by higher maintenance costs, equipment replacement, and additional workforce training requirements (41). Therefore, the long-term benefits of infrastructure investment depend not only on acquisition of new assets but also on the availability of sustainable financing mechanisms to support their maintenance and effective use throughout their operational life cycle (42).

### 4.5 Limitations

Several limitations should be considered when interpreting the findings.

First, this study examined the financial dynamics of a single public multidisciplinary hospital. Although the institution is a major provider of specialized healthcare services, its financial profile may not be representative of hospitals operating under different organizational models, financing arrangements, or regional conditions. Therefore, the findings should be generalized with caution.

Second, the analysis was based on audited annual financial statements. Although these reports provide standardized and reliable information, they do not capture short-term fluctuations, seasonal variation, temporary liquidity pressures, or other within-year changes that may influence financial performance. More frequent financial reporting could provide a more detailed picture of institutional financial dynamics.

Third, the available financial reports did not include the complete balance-sheet structure. Consequently, some ratios were derived from selected financial indicators rather than the full set of accounting variables and should therefore be interpreted as descriptive measures of financial dynamics rather than precise indicators of overall financial performance or solvency.

Fourth, the observation period was limited to five annual reporting cycles. Although this timeframe was sufficient to describe financial changes during 2020–2024, it does not allow assessment of longer-term structural trends or determination of whether the observed patterns will persist over time.

Fifth, the study relied exclusively on financial reporting data and did not include information on service volume, case mix, staffing, reimbursement policies, managerial decisions, or other organizational factors that may have influenced the observed financial patterns. As a result, the relative contribution of these factors could not be evaluated. Accordingly, the observed financial changes should be interpreted within the context of the available financial data, as the study did not examine the contribution of individual organizational or policy factors.

Sixth, comparable financial data from other hospitals were not available. Consequently, it was not possible to benchmark the observed indicators against regional or national averages or to determine whether the identified trends were specific to the study hospital or reflected broader developments within the healthcare system.

Finally, this study focused on financial indicators and did not evaluate clinical outcomes, healthcare utilization, patient satisfaction, or quality of care. Therefore, changes in financial indicators should not be interpreted as direct evidence of changes in healthcare performance. Likewise, although the hospital operated under a public-private partnership model throughout the study period, the study design did not allow the independent effect of this governance arrangement to be assessed. Future multicenter studies integrating financial, operational, and clinical indicators across hospitals with different organizational models would provide a more comprehensive understanding of institutional performance and post-pandemic adaptation.

## 5 Conclusion

This study described changes in the financial indicators of a public multidisciplinary hospital in Kazakhstan during 2020–2024 using audited financial statements. Considerable variation was observed in receivables, payables, inventories, and liquidity during the pandemic years, followed by a more stable financial pattern after 2022. Despite these changes, the hospital maintained a balanced financing structure throughout the study period.

The findings demonstrate that routinely available financial indicators can provide useful information on institutional financial dynamics during periods of major healthcare system disruption. Regular analysis of these indicators may support financial monitoring and management in public hospitals.

As evidence from Central Asia remains limited, this study contributes new data on hospital financial performance during and after the COVID-19 pandemic. Further multicenter studies involving hospitals with different organizational and financing models are needed to better understand financial adaptation across the healthcare system.

## Data Availability

The raw data supporting the conclusions of this article will be made available by the authors, without undue reservation.
