# Supplementary material for: Financial dynamics and post-pandemic recovery of a public multidisciplinary hospital in Kazakhstan: a five-year financial case study
Source: Front Public Health. 2026 Aug 25;14:1902342. doi: 10.3389/fpubh.2026.1902342 (PMC13549886; doi:10.3389/fpubh.2026.1902342)
Supplement: Supplementary file 1 [file Table_1.DOCX]

**Supplementary Table S1.** Detailed Balance Sheet Indicators of Almaty Multidisciplinary Clinical Hospital, 2018-2024 (thousand KZT)

| **Indicator** | **2018** | **2019** | **2020** | **2021** | **2022** | **2023** | **2024** |
| --- | --- | --- | --- | --- | --- | --- | --- |
| Cash and cash equivalents | 5,003 | 2,332 | 97,254 | 375,376 | 89,627.0 | 110,919.3 | 225,770.6 |
| Accounts receivable | 119,424 | 106,160 | 463,934 | 236,584 | 198,455 | 338,180 | 434,491 |
| Inventories | 186,300 | 210,895 | 281,068 | 597,809 | 655,870 | 665,253 | 748,922 |
| Fixed assets | 1,826,154 | 3,220,245 | 3,032,827 | 2,665,257 | 2,675,983 | 2,618,221 | 2,970,876 |
| Total assets | 2,136,907 | 3,539,918 | 3,875,083 | 3,875,026 | 3,626,449 | 3,733,220.7 | 4,380,706.7 |
| Current liabilities | 582,551 | 1,011,778 | 1,002,790 | 766,372 | 770,341 | 644,691.1 | 1,294,533.8 |
| Long-term liabilities | 736,606 | 291,014 | 519,969 | 637,216 | 437,519 | 437,519 | 432,961 |
| Total liabilities | 1,319,157 | 1,302,792 | 1,522,759 | 1,403,543 | 1,207,860 | 1,082,210.1 | 1,727,494.8 |
| Equity | 817,750 | 2,237,126 | 2,352,324 | 2,471,438 | 2,418,589 | 2,651,010.6 | 2,653,211.9 |

*Abbreviations: KZT, Kazakhstan tenge.*

*Note. Data were extracted from the audited annual balance sheets (Form No. 1). All values are presented in thousand KZT.*

**Supplementary Table S2.** Annual Income Statement Indicators of Almaty Multidisciplinary Clinical Hospital, 2018-2024 (thousand KZT)

| **Indicator** | **2018** | **2019** | **2020** | **2021** | **2022** | **2023** | **2024** |
| --- | --- | --- | --- | --- | --- | --- | --- |
| Total revenue | 3,651,276 | 3,778,545 | 4,547,385 | 6,517,544 | 6,226,754 | 7,655,012.8 | 8,191,573.2 |
| Total expenditures | 3,580,312 | 3,357,360 | 4,505,782 | 6,133,001 | 5,941,262 | 7,422,595.2 | 7,958,004.7 |
| Net financial result | 70,964 | 421,185 | 41,603 | 384,543 | 285,492 | 232,417.6 | 233,568.5 |

*Abbreviations: KZT, Kazakhstan tenge.*

*Note. Data were extracted from the audited annual income statements (Form No. 2). All values are presented in thousand KZT.*

**Supplementary Table S3.** Annual Operational Indicators of Almaty Multidisciplinary Clinical Hospital, 2018-2024

| **Indicator** | **Unit** | **2018** | **2019** | **2020** | **2021** | **2022** | **2023** | **2024** |
| --- | --- | --- | --- | --- | --- | --- | --- | --- |
| Outpatient visits | Persons | 38,674 | 35,572 | 34,180 | 40,315 | 40,617 | 36,721 | 37,081 |
| Hospitalized cases | Cases | 13,459 | 12,132 | 11,924 | 14,144 | 11,232 | 12,929 | 13,720 |
| Surgical procedures | procedures | 8,316 | 7,589 | 6,670 | 7,256 | 6,743 | 7,011 | 7,496 |
| Average length of stay | Days | 8.2 | 8.2 | 7.8 | 8.7 | 8.1 | 7.8 | 7.8 |
| Hospital beds | Beds | 330 | 330 | 330 | 430 | 350 | 350 | 360 |
| Bed turnover | times/year | 40.8 | 36.8 | 36.1 | 34.5 | 32.6 | 36.9 | 39.2 |
| Bed occupancy | days/year | 332.2 | 302.8 | 282.7 | 265.1 | 269.8 | 286.6 | 297.3 |
| In-hospital mortality | % | 2.3 | 2.3 | 1.6 | 2.1 | 1.4 | 1.4 | 1.1 |

*\*
